# Deep brain stimulation of the central thalamus restores arousal and motivation in a zolpidem-responsive patient with akinetic mutism after severe brain injury

**DOI:** 10.1038/s41598-024-52267-1

**Published:** 2024-02-05

**Authors:** Hisse Arnts, Prejaas Tewarie, Willemijn van Erp, Rick Schuurman, Lennard I. Boon, Cyriel M. A. Pennartz, Cornelis J. Stam, Arjan Hillebrand, Pepijn van den Munckhof

**Affiliations:** 1 Department of Neurosurgery, Amsterdam Neuroscience, Amsterdam UMC, University of Amsterdam, Meibergdreef 9, 1105 AZ Amsterdam, The Netherlands; 2 Department of Neurosurgery, Radboud University Medical Center, Nijmegen, The Netherlands; 3 Department of Clinical Neurophysiology and Magnetoencephalography Center, Amsterdam Neuroscience, Amsterdam UMC, Vrije Universiteit Amsterdam, Amsterdam, The Netherlands; 4 Amsterdam Neuroscience, Brain Imaging, Amsterdam, The Netherlands; 5 Amsterdam Neuroscience, Systems and Network Neurosciences, Amsterdam, The Netherlands; 6 Department of Primary and Community Care, Centre for Family Medicine, Geriatric Care and Public Health, Radboud University Medical Centre, Nijmegen, The Netherlands; 7 Accolade Zorg, Bosch en Duin, The Netherlands; 8 Libra Rehabilitation & Audiology, Tilburg, The Netherlands; 9 Cognitive and Systems Neuroscience Group, Swammerdam Institute, Center for Neuroscience, University of Amsterdam, Amsterdam, The Netherlands

**Keywords:** Hypoxic-ischaemic encephalopathy, Disorders of consciousness

## Abstract

After severe brain injury, zolpidem is known to cause spectacular, often short-lived, restorations of brain functions in a small subgroup of patients. Previously, we showed that these zolpidem-induced neurological recoveries can be paralleled by significant changes in functional connectivity throughout the brain. Deep brain stimulation (DBS) is a neurosurgical intervention known to modulate functional connectivity in a wide variety of neurological disorders. In this study, we used DBS to restore arousal and motivation in a zolpidem-responsive patient with severe brain injury and a concomitant disorder of diminished motivation, more than 10 years after surviving hypoxic ischemia. We found that DBS of the central thalamus, targeted at the centromedian-parafascicular complex, immediately restored arousal and was able to transition the patient from a state of deep sleep to full wakefulness. Moreover, DBS was associated with temporary restoration of communication and ability to walk and eat in an otherwise wheelchair-bound and mute patient. With the use of magnetoencephalography (MEG), we revealed that DBS was generally associated with a marked decrease in aberrantly high levels of functional connectivity throughout the brain, mimicking the effects of zolpidem. These results imply that ‘pathological hyperconnectivity’ after severe brain injury can be associated with reduced arousal and behavioral performance and that DBS is able to modulate connectivity towards a ‘healthier baseline’ with lower synchronization, and, can restore functional brain networks long after severe brain injury. The presence of hyperconnectivity after brain injury may be a possible future marker for a patient’s responsiveness for restorative interventions, such as DBS, and suggests that lower degrees of overall brain synchronization may be conducive to cognition and behavioral responsiveness.

## Introduction

Zolpidem-induced neurological recoveries are a well-known, but relatively rare phenomenon after severe brain injury^1^. While some patients only experience a temporary effect of the drug, others may have consistent responses over years or build up a complexity of behaviors with repeated dosing^2–4^. For most patients, the ‘awakening’ effects of zolpidem, such as return of movement, speech, or other cognitive functions are relatively short-lived, only lasting for a couple of minutes or hours, possibly related to the relatively brief duration of peak concentration in the blood after administration of the drug^5,6^. Moreover, there is a significant wearing-off effect, often resulting in a situation wherein zolpidem can only be given on a couple of consecutive days with a subsequent ‘drug-holiday’ that remains necessary for the drug to regain its positive initial effects. The fact that zolpidem reveals a tantalizing glimpse of residual brain functions in patients with severe neurological deficits can provide hope, and, at the same time, be extremely frustrating for families and caregivers. For now, there are few explanations for the drug-tolerance that develops with repeated zolpidem administrations and there are no permanent solutions to restore residual brain functions in this enigmatic group of patients.

Previously, we described a patient with akinetic mutism, a severe disorder of diminished motivation characterized by a loss of spontaneous motor function and speech, but with presence of intact consciousnesss, who showed spectacular signs of ‘awakening’ after zolpidem, including a short-lived return of walking and talking, more than 10 years after hypoxic-ischemic brain injury. Using magnetoencephalography (MEG), we showed that this zolpidem-induced recovery was associated with a temporary normalization of pathologically increased levels of functional connectivity in the brain^5^. Since the effects of zolpidem were short-lasting (approximately one hour) and, because of drug tolerance, zolpidem could only be given on specific occasions, the search for further treatment options continued. Following up on the MEG findings, we hypothesized that other treatments capable of lowering the high levels of functional connectivity would be a key to more permanently restore residual brain functions^3^. Deep brain stimulation (DBS) was considered as an experimental last-resort treatment option. DBS is a well-established technique that is associated with the modulation of neural activity and connectivity through the administration of small bouts of electric currents with electrodes that are surgically placed in specific brain targets^7^. Previous studies of DBS in other neurological conditions, such as in Parkinson’s disease, showed that DBS is able to modulate functional connectivity throughout the brain and, thereby, can influence disease-specific patterns of oscillatory activity^8,9^. Moreover, there is already a relatively large body of historical and more recent experimental evidence that DBS of the central thalamus can influence arousal regulation and behavioral performance, which is an effect that could translate well to our patient with akinetic mutism^10–12^. DBS of both the centromedian-parafascicular complex (CM-Pf) and centrolateral nucleus (CL) of the thalamus have previously safely been performed in both healthy anesthesized nonhuman primates, as well as humans with disorders of consciousness^13–16^. Though its working mechanism remains unknown, some authors have suggested that DBS, similarly to zolpidem, restores signaling in the so-called ‘mesocircuit’ that is believed to be involved in regulation of (forebrain) arousal and to be dysfunctional after brain injury, especially in those with disorders of consciousness^17–19^. The thalamus is centrally positioned in this mesocircuit and the nuclei of the central thalamus are interposed between brainstem arousal systems and frontal cortical attentional systems^20^. Stimulation of this structure, with its widespread cortical connections, may, similarly as in patients with disorders of consciousness, restore frontal-subcortical signaling in our patient with akinetic mutism.

In this explorative study, we studied both the clinical and neurophysiological effects of DBS of the central thalamus (primarily targeted at the CM-Pf complex)^21^. The aim of this study was to improve the clinical condition of the patient more than 10 years after experiencing hypoxic ischemia. Moreover, we used magnetoencephalography (MEG) to study the underlying neurophysiological effects of DBS on functional connectivity throughout the brain.

## Materials and methods

### Ethical approval

The legal representatives of the patient gave written informed consent for this treatment. The ethical justification for the associated N = 1 protocol was evaluated by a multidisciplinary medical-ethical board that was specifically composed for this case, in which clinicians from all related specialties were represented along with an independent medical-ethical council. Our institutional clinical medical ethical committee (AmsterdamUMC) approved the compassionate use treatment. Moreover, written informed consent from the family was obtained for publishing information/images/videos in an online open-access publication. Ethics review criteria conformed to the Declaration of Helsinki. No part of the study procedures or analysis plans was pre-registered in an institutional registry prior to the research being conducted.

### Clinical case

A 29-year old male with a history of alcohol abuse suffered hypoxic-ischemic brain injury after choking on a piece of meat (this case is also previously described in Arnts et al.^5^). After apparent initial, though slow and modest neurological recovery, spontaneous movement and speech disappeared in a time-frame of about two to three weeks. The patient developed such a severe impairment of arousal that he required intensive auditory and tactile stimulation to maintain a wakeful state. No structural lesions were found using a computerized tomography (CT) scan to explain this secondary deterioration, and conventional EEG-recordings showed no evidence of interictal or ictal epileptiform discharges. After a stay in the ICU and neurology department, the patient was transferred to a nursing home without a formal diagnosis explaining his hyporesponsive state. A structural MRI at follow-up showed signs of diffuse atrophy without hydrocephalus.

Eight years passed without any further improvement or neurological follow-up. Upon clinical assessment, the now 37-year old patient seemed awake and maintained his eyes open, selectively followed people through the room, but showed a complete lack of voluntary movement (akinesia) and absence of speech (mutism). More specifically, the patient showed no affective reactions, initiation of eating or drinking, and remained incontinent. Although the patient exhibited no signs of spontaneous speech or vocalization on request, he was inconsistently able to respond to commands through delayed movement with evident ataxia and muscle rigidity. Despite his intact awareness, the patient’s initiative was so severely impaired that he remained wheelchair-bound and entirely dependent on nursing care for all daily activities, including the need for enteral tube feeding. His behavioral condition was classified as akinetic mutism: a severe disorder of diminished motivation^21^. The patient reacted quite repelling when being touched in his face (washing and, especially, tooth brushing) and body (washing, dressing). Nursing care of daily activities therefore caused considerable discomfort. Medical treatments such as a visit to the dentist or changing the enteral tube were hardly feasable.

As previously described, the patient showed a rather spectacular response to administration of zolpidem (10 mg), a sleeping-drug that is frequently used for patients with persistent hyporesponsive disorders^1^. Typically, within 20 min, the patient would start communicating spontaneously, eat by himself and, at times, even managed to walk while being supported. Despite having amnesia and a hearing deficit, he would be rather cheerful, alert, and would show interest in the people and objects around him. He was collaborative during nursing care, and medical appointments took place without incidents. However, this paradoxical reaction to zolpidem was relatively short-lived, usually lasting for about two hours, after which he would gradually fall back into his state of diminished arousal and motivation. Moreover, a severe reduction in effectiveness was noticeable after administering zolpidem for several consecutive days. The time windows during which the patient was able to talk and move became shorter, and his abilities to move and speak during these time-windows decreased. Usually, the restorative effects of a single dose of zolpidem could be reproduced once a day for about five consecutive days. After this period, drug administration did not result in observable clinical effects. The use of multiple doses of zolpidem during a single day showed no improvement in his clinical condition and sometimes even caused sedation. On average, it would require two to three medication-free weeks to notice the effects of a single dose of zolpidem again. Consequently, zolpidem administration was restricted to special occasions, such as family visits and medical appointments, which prompted family and medical staff to seek for further therapies to improve his motivational state and restore his purposeful behavior. The relatives stressed the medical staff to do ‘everything’ in order to improve the patient’s situation.

During a moral case deliberation with a multidisciplinary medical-ethical board, it was discussed that continuation of the current course of action would not increase the patient’s capacities of purposeful behavior or emotional expression, nor reduce the considerable discomfort related to activities of daily living. If DBS would enable him to eat by himself and actively participate in his daily care, this would considered as a real clinical value for the patient and as beneficial for his daily care by the healthcare staff. Moreover, if the patient could speak again or show some purposeful behavior on request, his treatment could possibly be further personalized to his individual wishes. Additionally, if he would display significant long-lasting progress, rehabilitative therapies could possibly be offered. On the other hand, what if he improved to an extent that he could become more aware of his despairing situation, yet remained severely disabled? Could he give informed consent himself for the procedure after receiving a dose of zolpidem? Based on the outcome of the deliberation, and approval of the multidisciplinary medical-ethical board, it was concluded that the research team would approach the surrogates (relatives) of the patient to discuss enrolling him as subject in a clinical DBS trial. It was also decided to try to deduce a ‘yes’ or ‘no’ answer from the patient after administration of zolpidem, though he did not show enough disease insight to conduct proper informed consent. Finally, It was decided not to continue treatment or research if any physical or other signs of objection were present. The reversibility of DBS was mentioned to partly justify the start of the DBS trial, since potential negative effects could presumably be reversed.

At the start of the current study, the patient was 40 years old. During the study period, the patient received no zolpidem or other drugs or neurostimulants that could have affected his neurological condition.

### Clinical assessment

Clinical tests were performed before and during DBS using the Montreal cognitive assessment tool (MoCA), a brief screening tool used for patients with mild cognitive impairment^22,23^. Tests could only be performed at irregular intervals due to the COVID-19 pandemic restrictions.

### DBS surgery

Before surgery, the patient underwent a 3 T stereotactic MRI scan (Siemens, Malvern, Pennsylvania, USA), including axial T2-weighted and post-gadolinium (Gd) volumetric axial T1-weighted sequences. Pre-operative CM-Pf targets were determined from the mid-commissural point on anterior–posterior (AC-PC) aligned MRI images. Target planning for the central thalamus (intentionally aimed at the CM-Pf complex using traditional stereotactic methods) was optimized, based on the width of the third ventricle with final coordinates: 9.8 mm lateral, 9.5 mm posterior, and 2.8 mm ventral to the midcommissural point. Planned trajectories were inspected to be pre-coronal, start on top of a gyrus, and to avoid ventricles and blood vessels.

**Figure 1:**
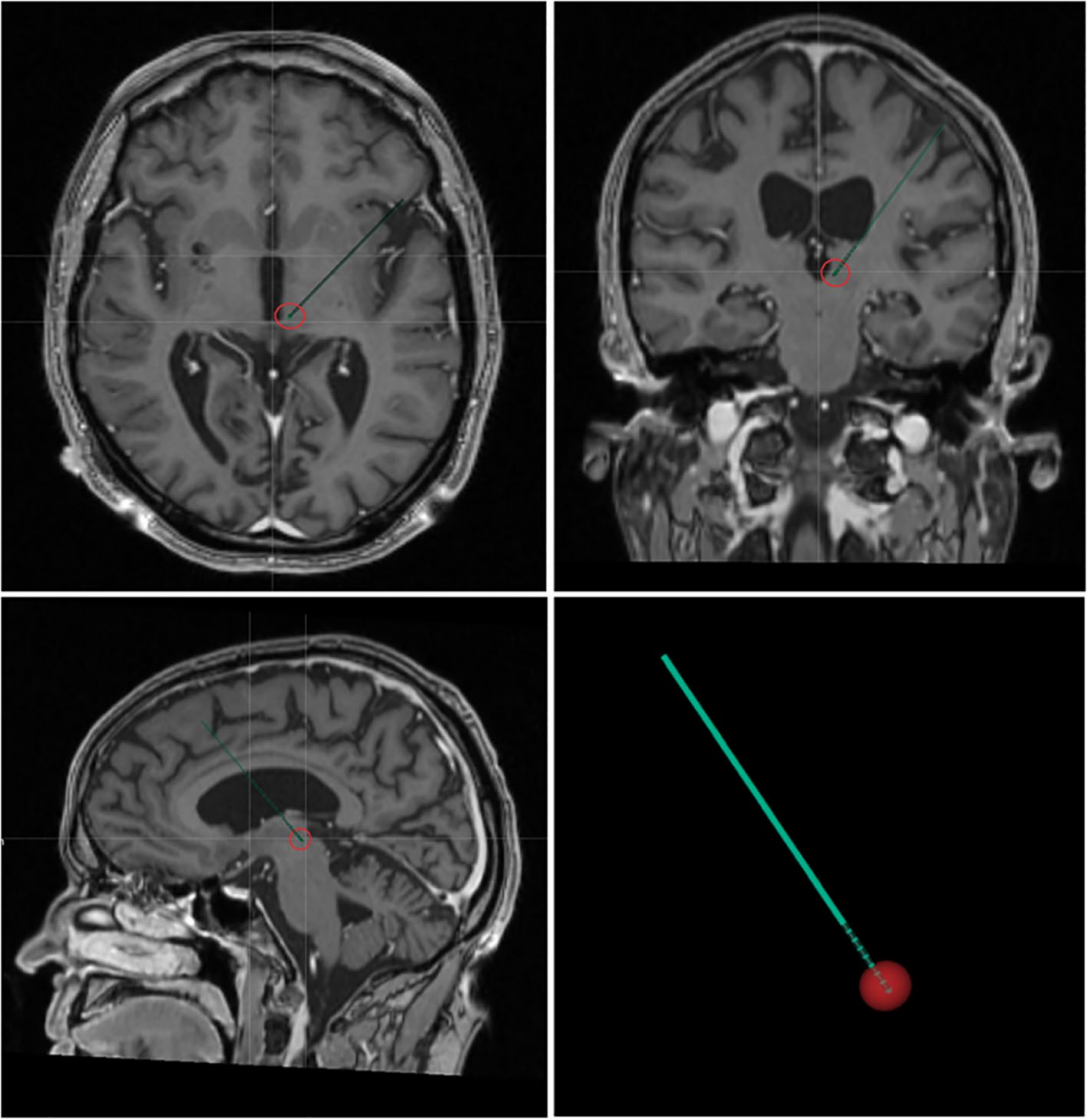
Postoperative lead localization of the right DBS electrode projected in a T1-weighted MRI, showing active contact points in the centromedian-parafascicular complex with its effective electrical field size visualized (bottom right) by calculating the volume of tissue activated in Brainlab’s Guide-XT software module (Brainlab AG, Munich, Germany, version 3.2.0.281). Monopolar settings were used with the lowest contact point as cathode and pulse generator as anode.

On the day of the surgery, a Leksell stereotactic frame (G-model, Elekta Ab, Stockholm, Sweden) was placed under general anesthesia and the patient was transported to the 1.5 T MRI, where a frame-based stereotactic MRI was obtained. After fusion with the pre-operative 3 T MRI using Brain Elements software (Brainlab AG, Munich, Germany, version 3.2.0.281) stereotactic coordinates of the planned targets were obtained. The patient was returned to the operating room and burr holes were made. A rigid macrostimulation electrode (Elekta) was inserted into the left and right target and replaced by a Boston Vercise™ Cartesia lead with eight 1.5 mm contact points separated by 0.5 mm interspaces (model DB2202, Boston Scientific, California, USA) under fluoroscopy. Left-sided ventral-to-dorsal contacts encoded points 1–8 and right-sided ventral-to-dorsal contacts encoded points 9–15. During the same surgical session, a corresponding Boston Vercise™ pulse generator was implanted in a subcutaneous pocket in the infraclavicular region under general anesthesia. One day after the operation, a CT-scan was made and co-registered to the MRI for lead localization (see Fig. 1 for electrode localisation of the right DBS elektrode). The patient was discharged two days after surgery, with DBS still off. After two weeks, the first stimulation session was started (see “Clinical effects of DBS”). Monopolar settings were used during the study on both the left and right DBS electrode, with the lowest contact point as cathode and pulse generator as anode.

### MEG

#### Recordings

The MEG recordings (22 months after the operation) were obtained in a magnetically shielded room in a supine resting-state condition. MEG data were recorded using a 306-channel Triux Neo whole-head system (MEGIN, Helsinki, Finland) with a sampling frequency of 2000 Hz, online anti-aliasing (410 Hz) and high-pass filtering (0.1 Hz), and internal active shielding (IAS) off. The head position relative to the MEG sensors was recorded continuously using the signals from five head position indicator (HPI) coils. The HPI positions and the outline of the patient's scalp (around 2000 points) were digitized before the MEG recording using a 3D digitizer (Fastrak, Polhemus, Colchester, VT, USA). The patient's MEG data were co-registered to the structural MRI, using a surface-matching procedure with an estimated resulting accuracy of 4 mm^24^. This structural T1-weighted MRI of the head had been obtained before the baseline MEG session as part of clinical care, using a 3 T Siemens MRI scanner (Siemens, Malvern, Pennsylvania, USA).

Pre-DBS, MEG data from this patient had already been recorded as a reference for further research. Twelve months after implantation of the electrodes, multiple post-DBS MEG datasets were recorded in a single session, starting with a 10 min eyes-closed resting-state condition without stimulation (the DBS off condition). The stimulator had been off for 12 h. Hereafter, the DBS was switched on for 10 min in the MEG, using its usual 50 Hz, 450 μsec, 2.3 mA) parameters. The DBS caused significant artifacts in MEG-signal (similar as described in Arnts et al.^12^). Therefore, it was decided to turn the DBS off and record a wash-out period of another 10 min in which the patient was still aroused and maintained the effects of stimulation (in figures: ‘DBS effect’). Hereafter, a 5-min break followed, after which the stimulator was switched back on for another recording of 10 min. Finally, another wash-out period without stimulation was recorded with a similar length of time.

##### Pre-processing and source reconstruction

The MEG data were first cleaned using both spatial and temporal filtering, after which the sensor-level data were projected to source-space using an atlas-based beamformer^25^. Neuronal activity (relative power and variability) and functional connectivity were quantified at the source-level. In more detail, bad channels were first removed after visual inspection of the data (by co-author PT). Thereafter, the temporal extension of Signal Space Separation (tSSS) in MaxFilter software (Elekta Neuromag Oy, version 2.2.15) was applied using standard settings: a correlation limit of 0.9, and a sliding window of 10 s^26,27^. The automated anatomical labeling (AAL) atlas was used to label the voxels in 78 cortical and 12 subcortical regions of interest (ROIs)^28,29^. This was done by registering the anatomical T1-weighted image to an MNI template and labeling all voxels according to the 90 ROIs. Subsequently, an inverse registration to anatomical subject space was performed. We used each ROI's centroid voxel as a representative for that ROI^30^. Subsequently, a scalar beamforming approach (beamformer, version 2.1.28; Elekta Neuromag Oy), similar to Synthetic Aperture Magnetometry, was used to project the sensor-level data to these centroids^31^. The beamformer weights were based on the covariance of the recorded time-series within a 0.5–48 Hz frequency window and the forward solution (lead field) of a dipolar source at the centroid voxel location, and using a single sphere head model fitted to the MRI scalp surface as extracted from the co-registered MRI^32,33^. Source orientation that maximized the beamformer output was obtained using eigenvalue decomposition^34^. Singular value truncation was used when inverting the data covariance matrix to deal with the rank deficiency of the data after SSS (∼ 70 components). Broadband data (0.5–48 Hz) were projected through the normalized beamformer weights^30,35^, resulting in a broadband time series for each centroid of the 90 ROIs^30,35^.

The amount of data used for further analysis, per condition, was determined by the amount of artifact-free data (based on visual inspection by co-author PT). For analysis of stimulation, we used the MEG dataset during the washout phase, in specific, the first minutes directly after cessation of stimulation. MEG-based functional connectivity (see below) for the patient was compared with the average functional connectivity obtained from healthy volunteers. Based on gender and age, we selected out of a previously published dataset of healthy volunteers all males of approximately the same age as this patient^30,36^. This resulted in three healthy age-matched males (mean age of 39), who had all undergone one five-minute, eyes-closed, resting-state MEG recordings (similar to Arnts et al.^5^). Data acquisition, pre-processing, and analysis was performed in the same way as for the patient’s reference dataset.

##### Estimation of spectral power, functional connectivity and neural variability

We estimated power spectral densities using a Fast Fourier Transform (FFT) using Welch’s method, with window width of 10 s. Power spectral densities were averaged over all ROIs and artefact free epochs (range 23–30 epochs). We defined frequency bands as follows: theta (4–8 Hz), alpha1 (8–10 Hz), alpha2 (10–13 Hz), beta (13–30 Hz), and gamma (30–48 Hz). Functional connectivity was estimated using the amplitude envelope correlation (AEC) from band-pass filtered time-series^37^. The AEC captures co-fluctuations in modulations of the amplitude envelope. Pairwise orthogonalization was first performed to reduce the effects of signal leakage prior to connectivity estimation^38,39^. The amplitude envelopes were extracted from the analytical signal obtained from the Hilbert transform for every band-pass filtered time series. No further smoothing or downsampling was applied to the amplitude envelopes. We computed the AEC using the same epoch length as used for the power spectral densities (10 s). Pearson correlations between amplitudes envelopes were computed. The AEC was calculated for all possible pairs of ROIs, resulting in a 90 × 90 weighted adjacency matrix that contained the functional connectivity values between all pairs (with a potential range of values between − 1 and 1). Averaging over rows in this weighted adjacency matrix subsequently led to one mean functional connectivity value per ROI (i.e. the average functional connectivity of that ROI with the rest of the brain) per condition.

Past work has shown that fluctuations in the amplitude envelope coincide with strong functional connectivity, i.e. periods of high amplitude envelope could serve as a window of opportunity for ongoing functional connectivity^40^. Hence, as the AEC captures co-fluctuations in the amplitude envelope, we lastly estimated the variability of the amplitude envelopes in the context of neural variability^41^. Neural variability was quantified in terms of the detrended standard deviation of the amplitude envelope for every ROI. We first subtracted the mean from every time series for every ROI, after which we computed the standard deviation for each epoch of 10 s^42^. The lower limit for the detrended standard deviation is zero and the upper limit is determined by the range of the values for every time series. Power spectral density, neural variability, and functional connectivity analyses were performed in MATLAB 2021b (Mathworks; 9.1.0.441655) using in-house scripts. The observed power and AEC for different conditions are reported as average and standard deviation across epochs. These standard deviations across epochs are depicted as error-bars in the figures in the result section. We assumed that differences between groups could not be obtained by chance if there was no overlap between the error-bars. However, caution should be taken with formal statistics for this n = 1 study.

## Results

### Clinical effects of DBS

At the start of the study, the baseline MOCA-score was 0/30. DBS with high-frequency parameters (130 Hertz (Hz), 60 microseconds (μs), 1 milliampere (mA)) elicited a direct arousal response: the eyes of the patient were widened and there were signs of active visual pursuit throughout the room with motor restlesness in his weelchair. However, no objective signs of return of spontaneous speech or purposeful movements could be observed. It was decided to leave the stimulator on for 24 h. Hereafter, the patient was repeatedly visited in his nursing home to evaluate his clinical condition. However, after 2 months of stimulation, still no signs of return of speech or movement could be seen. The MOCA-score remained 0/30. Several parameter changes were made within the high-frequency mode, including an increase in amplitude and pulse width, but some of these changes gave signs of discomfort, possibly by spreading of current to the sensory thalamus in the neighbourhood of the CM-Pf, causing the patient to feel tingling. After this period, a parameter switch was made to low-frequency stimulation (30 Hz, 450 μs) following our observations in another patient who received DBS for a disorder of consciousness following traumatic brain injury^12^. Low-frequency stimulation, similar to high-frequency stimulation, resulted in a direct increase in arousal, motor restlessness and visual pursuit (see Video 1). Hereafter, slow, but progressive signs of improvement in behavioral performance followed. For instance, it was observed that the patient showed consistent communication with the use of a letter map and needed less additional enteral feeding, since eating was improved (i.e. he ate by himself). His MOCA score increased to 2/30, though these improvements were still smaller than those previously observed with zolpidem administration (maximum MOCA score of 13/30). Therefore, it was decided to increase the amplitude of continuous stimulation to 1.5 mA. Two months later, he clearly named three animals in the MOCA test (score of 3/30), and, at times, ate autonomously with some verbal stimulation. During this time, his nursing team noticed a decrease in length of sleep and in days with excessive tiredness. Therefore, it was decided to switch the stimulator off at night and on in the morning. After further raising the current to 2.3 mA, our patient could now be woken up in the morning from a state of sleep (Video 2). Moreover, the patient could now, at times, eat and drink by himself (Video 3). The day after raising the amplitude to 2.3 mA, the patient was even able to walk with a walker, communicate with family and staff, make jokes, actively watch TV, and make requests for food that he would like (Video 4). These improvements in behavioral performance were clearly better than those previously observed with zolpidem. His MOCA score improved to 6/30. However, after two days of improvement, these spectacular effects disappeared and excessive tiredness and immobility reappeared. The DBS settings were kept constant for several months to see if spontaneous improvements or ‘up-regulation’ would occur, but without success. Therefore, it was decided to increase the stimulation amplitude to 2.6 mA. Once again, a spectacular awakening occurred after a couple of hours with return of talking, walking and spontaneous communication with healthcare staff. His MOCA score further increased to 11/30, even with some return of mathematical abilities. However, the same night, the patient became increasingly confused, requesting his healthcare staff to ‘bring him home’, eventually becoming angry with staff and family members (on the phone) and, at times, feeling sad. The day after, this confused state disappeared, slowly changing to a state of extreme tiredness. Therefore, after careful deliberation with both family and healthcare staff, it was decided to reduce the amplitude to 2.3 mA, as this confused state and tiredness were deemed not to be in the best interest of the patient. With 2.3 mA of daytime stimulation, a stable situation developed in which the patient required less enteral feeding, was able to sometimes eat by himself, and, at times, could assist in his own self care. Signs of confusion and sadness were not observed anymore. Later, a frequency change to 50 Hz was made, in an attempt to increase the lengths of daytime arousal and periods with increased behavioral performance. In order to record the neurophysiological changes, and gain insights in possible mechanisms underlying the effects of DBS, it was decided to perform another MEG recording. The MEG recording took place 22 months after the DBS operation, and the patient was stimulated with DBS settings of 50 Hz, 450 μs, and 2.3 mA.

### MEG results

**Figure 2:**
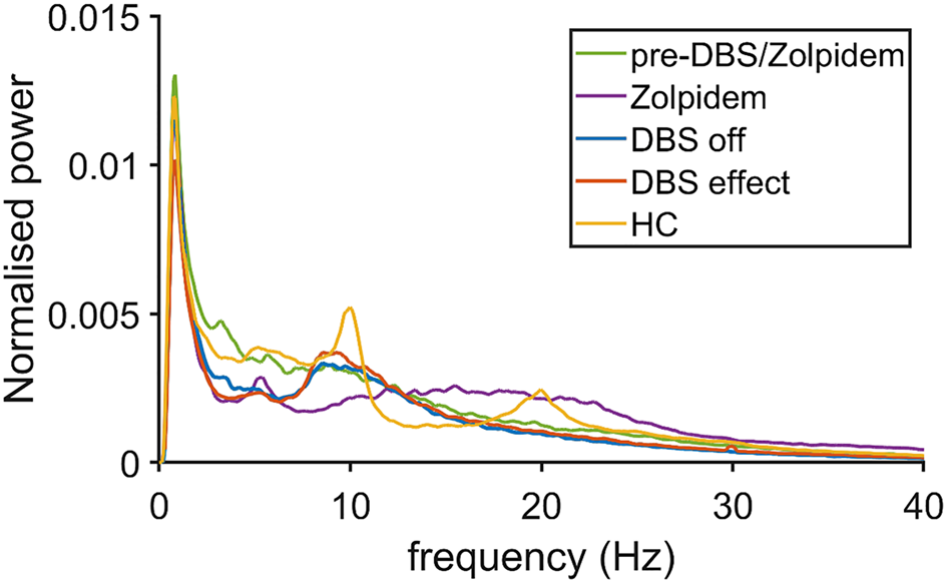
Spectral analysis of four different conditions and healthy controls (grand average over all regions). Green: pre-DBS/zolpidem, purple: after zolpidem administration, red: DBS off condition, blue: DBS effect, yellow: healthy controls (HC).

The MEG source-space spectral analysis showed differences in spectral power between our patient and healthy controls (see Fig. 2). Previous research already showed a specific decrease in the alpha-peak and increase in beta-band power in our patient after zolpidem administration. However, after DBS, rather limited changes were seen in the power spectrum throughout the brain. Low-frequency DBS showed some limited but notable decrease in theta-band power and increase in alpha-band power compared to the DBS off condition, although the alpha-peak in healthy controls remained more prominent (see Supplementary Material, Fig. 1).

**Figure 3:**
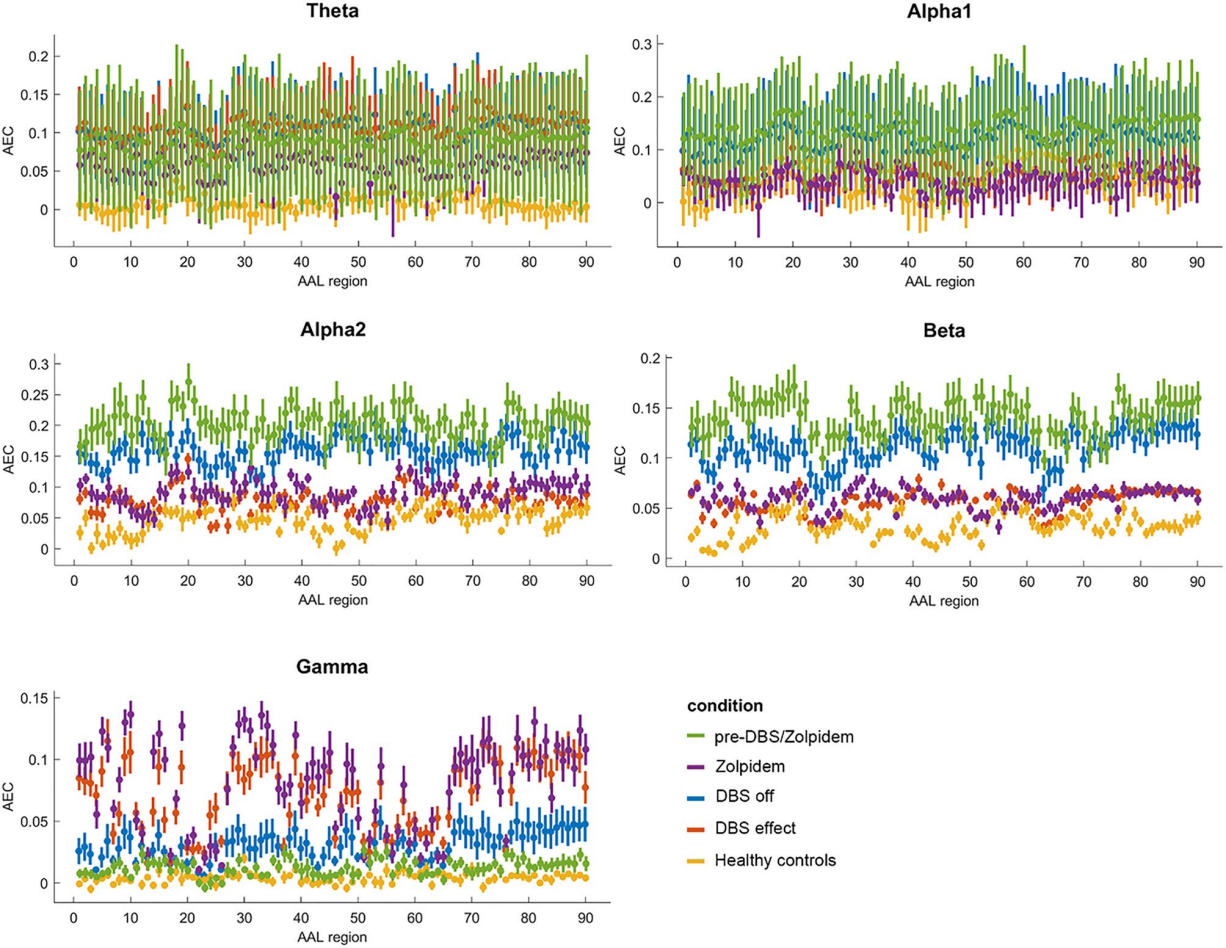
Regional functional connectivity of four different conditions and healthy controls. Green: pre-DBS/zolpidem, purple: after zolpidem administration, red: DBS off condition, blue: DBS effect, yellow: healthy controls. For definition of AAL regions: see Supplementary Table 1. Note: error bars depict ‘confidence intervals’ based on the standard deviation of each computed metric (measured within epochs/time) in each condition.

**Figure 4:**
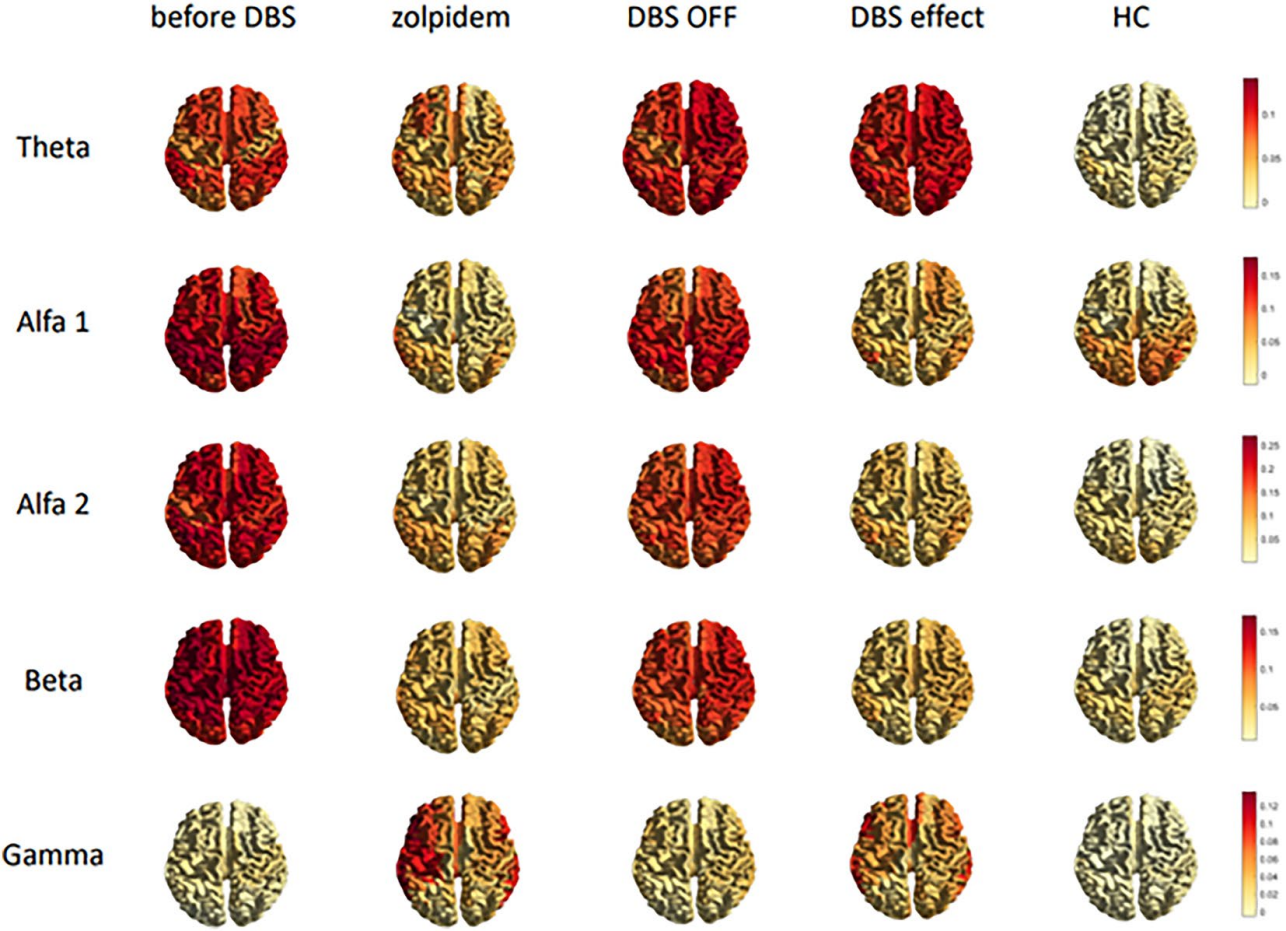
Regional differences in functional connectivity of four different conditions compared to healthy controls (darker red = regions with high levels of functional connectivity, light-yellow = regions with lower levels of functional connectivity).

There were, however, striking differences in functional connectivity after low-frequency (50 Hz) DBS (see Fig. 3). First of all, there were remarkable decreases in functional connectivity with respect to the DBS off condition for almost all regions, with the alpha1-band, alpha2-band, and beta-band approaching baseline levels of functional connectivity that were measured in healthy controls. The direction and magnitude of these changes were similar to the changes previously seen after zolpidem administration. Moreover, there were increases in functional connectivity within the gamma-band, similar as seen after zolpidem administration, although these levels were markedly higher than in healthy controls. The analysis of patterns of regional functional connectivity for the different conditions showed no specific regions of interest, suggesting that these changes were widespread throughout the brain, though some areas of the brain, such as the visual cortex, showed no changes in gamma-band functional connectivity after DBS (see Fig. 4). Importantly, there was a large variation in the theta- and alpha-1 band for the situation before DBS and before zolpidem, suggesting that DBS and zolpidem result in some ‘stabilisation’ or limit variation.

**Figure 5:**
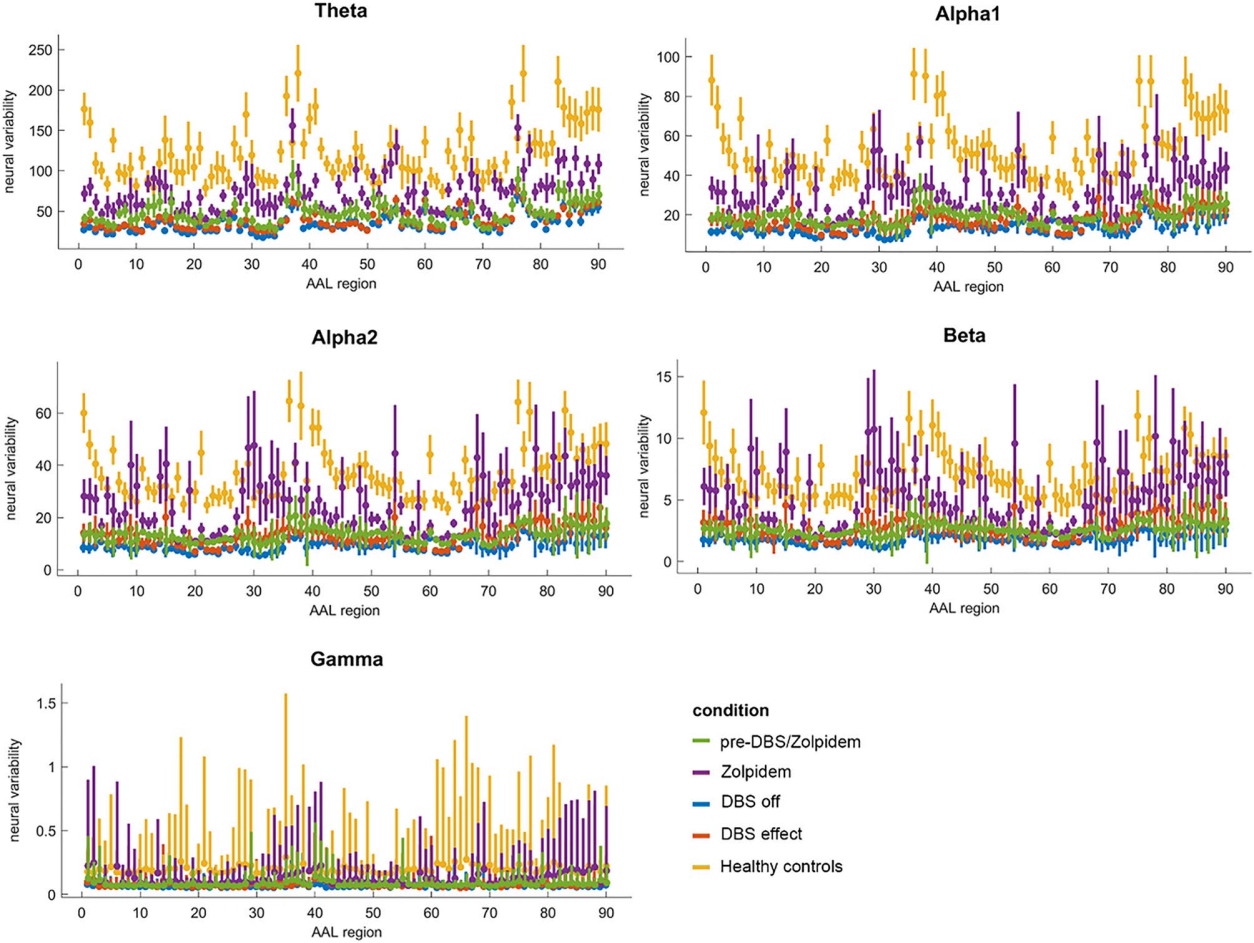
Neural variablity for four different conditions and healthy controls. Blue: DBS off condition, red: DBS effect, yellow: healthy controls, purple: after zolpidem administration, green: before DBS/zolpidem (baseline condition). For definition of AAL regions: see Supplementary Table 1. Note: error bars depict ‘confidence intervals’ based on the standard deviation of each computed metric (measured within epochs/time) in each condition.

Concerning neural variability, there was a higher neural variability in healthy controls compared to our patient in the DBS off condition for all bands and almost all regions (see Fig. 5). After DBS, no increase in neural variability was observed and there were no differences between the DBS off and on condition. This stands in contrast to the condition after zolpidem administration (before the DBS operation), in which an increase in neural variability could be seen in all frequency bands^2^.

## Discussion

### General discussion

In this study, we show that central thalamic DBS after severe brain injury is associated with beneficial clinical effects in arousal and is able to transform a patient with akinetic mutism from a state of sleep and immobility to full wakefulness. Although longer periods of neurostimulation were associated with improvements in spontaneous speech, movement, and restored functional abilities, such as independent walking and eating, these changes remained of a temporary nature and effects seemed to wear off. This, in some sense, mimicks the wearing-off effects that were previously seen after zolpidem administration. However, the effects of DBS were much more long-lasting than those observed after zolpidem administration, which were only lasting for roughly one hour. After one year of stimulation, DBS was associated with a sustained improvement in behavioral performance, although this improvement was still limited.

Although little differences were seen in the spectral analysis of the MEG, there was a mixture of effects of 50 Hz DBS on functional connectivity. Specifically, a reduction of functional connectivity could be observed in nearly all frequency bands, except the theta band. Moreover, lowering of the variation in theta- and alpha1-band functional connectivity were qualitatively observed. These MEG-observations suggest that DBS of the central thalamus has some similar effects on functional connectivity as zolpidem, mimicking its reduction of ‘hyperconnectivity’ throughout the cortex. Neurological disruption after brain injury is known to result in altered connectivity in large-scale neural networks. Hyperconnectivity may be a ‘paradoxical’ response to neurological disruption and has previously been described to occur in the early setting after traumatic brain injury^43^. It is thought to be a fundamental compensatory response of neural networks to provide the minimum resources to maintain an integral network, and meet task demands, and may be a sign of plasticity at work in the brain^44–46^. Usually, brain injury causes some combination of functional connectivity gain and loss that is expressed differentially throughout the network. The fact that there was still mainly hyperconnectivity throughout the brain in our patient, more than 10 years after brain injury, may suggest that there is pathological overcompensation and that the process of plasticity in this patient could not be completed or ‘went wrong’. Previous studies, especially in patients with disorders of consciousness, have shown focal or more widespread signs of hyperconnectivity throughout the brain in response to different types of injuries^47,48^. This study shows that reducing this long-lasting pathological hyperconnectivity may be one of the keys of restoring functional brain networks. Interestingly, experimental DBS of the same thalamic target area (CM-Pf) and concomitant restoration of arousal in another patient with severe brain injury was associated with an increase in functional connectivity^12^. Pre-existent levels of functional connectivity in this patient with another type of brain injury were, however, much lower than observed in our current patient, and even lower than those seen in healthy controls. The contrasting results of both studies therefore seem to indicate that DBS of the central thalamus can work two ways and restore functional connectivity towards a more healthy ‘baseline’. This baseline may be an important ‘steady-state’ for optimal information transfer and functionality of brain networks.

In contrast with the post-DBS changes that were observed for functional connectivity, no changes were seen in neural variability, which is a measure of the ability of the brain to adapt to rapid changes in cognitive demands and accommodate a high-dimensional space for coding a large varieties of information, as suggested by animal models^49–51^. Previous research has shown that severe brain injury is associated with a loss of neural variability, which corresponds to the low neural variability observed in our patient compared to healthy controls. Neural variability and a low level of neural correlations have been shown to be a much better correlate for behavior and wakefulness than traditional measures of neuronal oscillatory power^49,52,53^. The observation that, after central thalamic DBS, neural variability remained relatively unchanged and much lower than in healthy controls may indicate that, despite improvements in arousal, cognitive performance was still significantly disturbed. The contrast between the effects of DBS of the central thalamus on functional connectivity and neural variability, in combination with the limited long-term improvements in behavioral performance, suggests that DBS can re-activate dormant functional brain networks, but lacks the ability to fully improve those properties of those injured networks that serve cognitive capacities.

There are several explanations for the temporary effects of DBS in this case. The most probable explanation is that the combined non-selective activation of different elements of the intralaminar thalamus around the active contact points in this study may have caused indirect counteracting effects of stimulation. Studies in animal models found that there is a relative opposition of activation of CM-Pf and CL elements with DBS, and that key differences in synaptic physiology exist between, for instance, CL and Pf afferents within the striatum^14^. It is thought that not the CM-Pf, but the fibers traversing the CL are mainly responsible for the widespread (sub)cortical activation and enhancement of arousal and behavioral performance seen after DBS. It is hypothesized that CL activation leads to excitation of the medium-sized spiny neurons within the striatum and, thereby, causes disynaptic disinhibition of the thalamus. Co-activation of the CM-Pf could, in turn, downregulate this disinhibitory effect, since the Pf suppresses the medium-sized spiny neurons. Since the area between the CM-Pf and CL is rather small compared to the diameter of human DBS electrodes and its surrounding volume of tissue activated (VTA), there is a possibility that the DBS effects observed in this study stem from mixed effects within the central thalamus, or even beyond the thalamus itself, through anti-dromic activation of distant brainstem nuclei that are involved in arousal. The VTA used in this study is relative large (mainly originating from a pulse width of 450 μs), making co-activation of both CL and CM-Pf components likely. Despite increasing advances in image-guided DBS, for now, it remains impossible to visualize the individual nuclei and fiber tracts within the central thalamus with conventional MRI techniques, especially in a patient with thalamic atrophy. There still is a relatively large gap between what can be modeled and visualized in animals versus what is now possible in clinical practice. Targeting of the central thalamus in this study was performed with conventional stereotactic coordinates, derived from previous studies, and adapted to the patient’s individual anatomical landmarks. Further possible other explanations for the reduced effectiveness of DBS may be habituation (or ‘tolerance’) to stimulation through adaptation of the biological response of the stimulated neuronal network, which is also seen after DBS for other neurological disorders, such as in those patients that received DBS for essential tremor^54–56^. Moreover, the tissue response to chronically implanted electrodes, such as the response of (subcortical) microglia may be different in patients with a severely injured brain and thalamic atrophy, making gliosis around the electrodes a possible reason for a reduced stimulatory effect^56,57^. While a change in DBS parameters, such as alternating, cycling or ‘steering’ of stimulation has been suggested to deal with adverse or tolerance effects, these actions did not result in a more permanent improvement in our patient as of yet.

### Limitations

Since this is a N = 1 study, our findings should be interpreted with some caution. Firstly, the present study contains a more in-depth re-analysis of previously acquired MEG data (see Arnts et al.^5^). Since this new analysis had a broader focus, and was not limited to the beta-band, with use of longer epochs for analysis, additional decreases of functional connectivity in almost all frequency bands, apart from the theta-band, were found after zolpidem. It should be noted that in the time between both studies a new MEG-system was installed in our center. However, the hardware differences were minor and similar analytic methods and analytical pipeline were used. We therefore believe that any effect of this system-change on differences in outcome between the two studies is limited. No formal statistics were performed for this explorative n = 1 study. Secondly, as previously described, during 50 Hz stimulation, there were significant artifacts in MEG signal, limiting its direct analysis. Therefore, for the analysis of data following 50 Hz stimulation, we were obliged to perform the analyses on data that were recorded directly after cessation of the stimulation. These artifacts of DBS are well known in MEG research^58^. Previous studies have shown that the effects of stimulation can be reliably analyzed after cessation, and, because the effects of stimulation on arousal were relatively long-lasting, we think that this potential limitation is negligible^12,58^. During this study, mainly because of safety reasons, DBS parameters were used that are considered ‘normal’ settings in patients with other neurological disorders, such as in Parkinson’s disease (the high-frequency setting) and pain disorders (the low frequency setting). The differences between the two frequency settings observed in this study may simply be attributable to a larger VTA for the low-frequency setting (since we used a pulse width of 450 μs). Since the optional settings on the Boston DBS electrode model DB2202 are quite extensive, we have not used it to its whole spectrum yet. Finally, this study is subject to performance confounds, meaning that if the behavioral performance is substantially different in two experimental conditions, as is the case for the pre- and post-DBS conditions, some measures of brain physiology may also differ between those conditions, thereby representing an epiphenomenon. 

## Conclusion

DBS of the central thalamus can restore arousal and influence behavioral performance by restoring functional brain networks, long after severe brain injury. It is able to transition a patient with severe brain injury from a sleeping state towards full wakefulness and, temporarily, restore some signs of behavioral performance. Its underlying effects seem to have similarities with the paradoxical working-mechanism of zolpidem: both seem to be able to return functional connectivity levels towards a ‘healthier’ baseline, though lack the ability to fully restore the functional properties of brain networks so that permanent functions can return. Zolpidem-responsiveness may be a potential sign that there is ‘hidden’ brain capacity left after injury and could therefore be used as a potential marker for a patient’s responsiveness to any further restorative interventions, such as DBS. Future research on the role of hyperconnectivity after brain injury is necessary to understand more of this complex phenomenon.

### Supplementary Information

Supplementary Video 1.

Supplementary Video 2.

Supplementary Video 3.

Supplementary Video 4.

Supplementary Information.

## Data Availability

The MEG datasets used and/or analysed during the current study are available from the corresponding author on reasonable request.
