# Supplementary material for: Deep brain stimulation of the central thalamus restores arousal and motivation in a zolpidem-responsive patient with akinetic mutism after severe brain injury
Source: Sci Rep. 2024 Feb 5;14:2950. doi: 10.1038/s41598-024-52267-1 (PMC10844373; doi:10.1038/s41598-024-52267-1)
Supplement: Supplementary file 5 — Supplementary Information. [file 41598_2024_52267_MOESM5_ESM.docx]

**SUPPLEMENTARY MATERIAL**
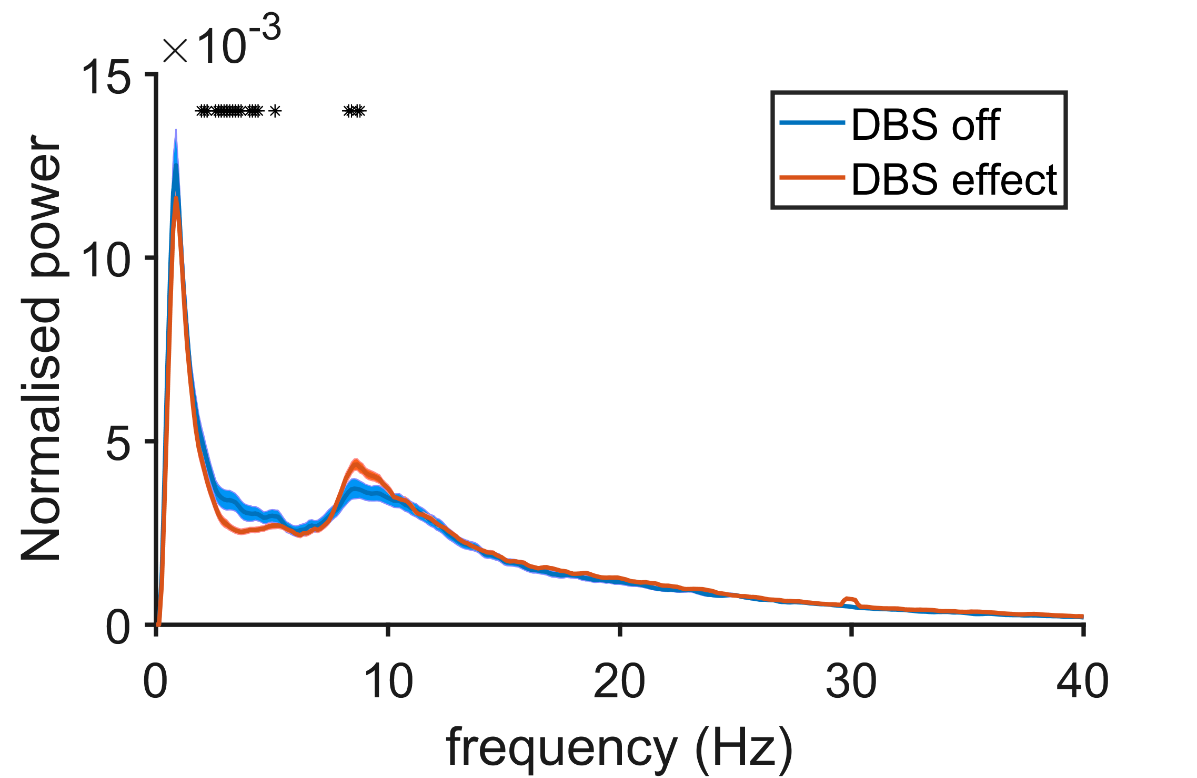


**Supplementary Figure 1**. Normalised power spectra (averaged over all regions) for the DBS OFF state and DBS effect with their respective 95% confidence intervals. * indicate frequencies for which the difference between the spectra for the two conditions was statistically significant (paired t-test; *p* < .05).

**Supplementary Table 1.** Regions of the AAL-atlas.

| **AAL atlas** | | | | | | | | |
| --- | --- | --- | --- | --- | --- | --- | --- | --- |
| **Frontal lobe** | | | | | | | | |
| 1 | Rectus_L | |  | | | 40 | | Rectus_R |
| 2 | Olfactory_L | |  | | | 41 | | Olfactory_R |
| 3 | Frontal_Sup_Orb_L | |  | | | 42 | | Frontal_Sup_Orb_R |
| 4 | Frontal_Med_Orb_L | |  | | | 43 | | Frontal_Med_Orb_R |
| 5 | Frontal_Mid_Orb_L | |  | | | 44 | | Frontal_Mid_Orb_R |
| 6 | Frontal_Inf_Orb_L | |  | | | 45 | | Frontal_Inf_Orb_R |
| 7 | Frontal_Sup_L | |  | | | 46 | | Frontal_Sup_R |
| 8 | Frontal_Mid_L | |  | | | 47 | | Frontal_Mid_R |
| 9 | Frontal_Inf_Oper_L | |  | | | 48 | | Frontal_Inf_Oper_R |
| 10 | Frontal_Inf_Tri_L | |  | | | 49 | | Frontal_Inf_Tri_R |
| 11 | Frontal_Sup_Medial_L | |  | | | 50 | | Frontal_Sup_Medial_R |
| 12 | Supp_Motor_Area_L | |  | | | 51 | | Supp_Motor_Area_R |
| 13 | Paracentral_Lobule_L | |  | | | 52 | | Paracentral_Lobule_R |
| 14 | Precentral_L | |  | | | 53 | | Precentral_R |
| 15 | Rolandic_Oper_L | |  | | | 54 | | Rolandic_Oper_R |
| **Parietal lobe** | | | | | | | | |
| 16 | Postcentral_L | |  | | | 55 | | Postcentral_R |
| 17 | Parietal_Sup_L | |  | | | 56 | | Parietal_Sup_R |
| 18 | Parietal_Inf_L | |  | | | 57 | | Parietal_Inf_R |
| 19 | SupraMarginal_L | |  | | | 58 | | SupraMarginal_R |
| 20 | Angular_L | |  | | | 59 | | Angular_R |
| 21 | Precuneus_L | |  | | | 60 | | Precuneus_R |
| **Occipital lobe** | | | | | | | | |
| 22 | Occipital_Sup_L | |  | | | 61 | | Occipital_Sup_R |
| 23 | Occipital_Mid_L | |  | | | 62 | | Occipital_Mid_R |
| 24 | Occipital_Inf_L | |  | | | 63 | | Occipital_Inf_R |
| 25 | Calcarine_L | |  | | | 64 | | Calcarine_R |
| 26 | Cuneus_L | |  | | | 65 | | Cuneus_R |
| 27 | Lingual_L | |  | | | 66 | | Lingual_R |
| **Temporal lobe** | | | | | | | | |
| 28 | Fusiform_L | | |  | | 67 | Fusiform_R | |
| 29 | Heschl_L | | |  | | 68 | Heschl_R | |
| 30 | Temporal_Sup_L | | |  | | 69 | Temporal_Sup_R | |
| 31 | Temporal_Mid_L | | |  | | 70 | Temporal_Mid_R | |
| 32 | Temporal_Inf_L | | |  | | 71 | Temporal_Inf_R | |
| 33 | Temporal_Pole_Sup_L | | |  | | 72 | Temporal_Pole_Sup_R | |
| 34 | Temporal_Pole_Mid_L | | |  | | 73 | Temporal_Pole_Mid_R | |
| 35 | ParaHippocampal_L | | |  | | 74 | ParaHippocampal_R | |
| **Insula and cingulate gyri** | | | | | | | | |
| 36 | Cingulum_Ant_L |  | | | 75 | | Cingulum_Ant_R | |
| 37 | Cingulum_Mid_L |  | | | 76 | | Cingulum_Mid_R | |
| 38 | Cingulum_Post_L |  | | | 77 | | Cingulum_Post_R | |
| 39 | Insula_L |  | | | 78 | | Insula_R | |
| **Central structures** | | | | | | | | |
| 79 | Hippocampus_L |  | | | 80 | | Hippocampus_R | |
| 81 | Amygdala_L |  | | | 82 | | Amygdala_R | |
| 83 | Caudate_L |  | | | 84 | | Caudate_R | |
| 85 | Putamen_L |  | | | 86 | | Putamen_R | |
| 87 | Pallidum_L |  | | | 88 | | Pallidum_R | |
| 89 | Thalamus_L |  | | | 90 | | Thalamus_R | |
